# A High-Efficiency Wideband Grating Coupler Based on Si_3_N_4_ and a Silicon-on-Insulator Heterogeneous Integration Platform

**DOI:** 10.3390/ma17040947

**Published:** 2024-02-18

**Authors:** Meng Liu, Xu Zheng, Xuan Zheng, Zisu Gong

**Affiliations:** College of Physics and Electronics Engineering, Qilu Normal University, Jinan 250200, China; zhengxu_sy@163.com (X.Z.); zhengxuan66@163.com (X.Z.); zisugongsdu@163.com (Z.G.)

**Keywords:** grating coupler, FDTD, coupling efficiency, wideband

## Abstract

To fully utilize the advantages of Si_3_N_4_ and Silicon-On-Insulator to achieve a high-efficiency wideband grating coupler, we propose and numerically demonstrate a grating coupler based on Si_3_N_4_ and a Silicon-On-Insulator heterogeneous integration platform. A two-dimensional model of the coupler was established and a comprehensive finite difference time domain analysis was conducted. Focusing on coupling efficiency as a primary metric, we examined the impact of factors such as grating period, filling factor, etching depth, and the thicknesses of the SiO_2_ upper cladding, Si_3_N_4_, silicon waveguide, and SiO_2_ buried oxide layers. The calculations yielded an optimized grating coupler with a coupling efficiency of 81.8% (−0.87 dB) at 1550 nm and a 1-dB bandwidth of 540 nm. The grating can be obtained through a single etching step with a low fabrication complexity. Furthermore, the fabrication tolerances of the grating period and etching depth were studied systematically, and the results indicated a high fabrication tolerance. These findings can offer theoretical and parameter guidance for the design and optimization of high-efficiency and broad-bandwidth grating couplers.

## 1. Introduction

In the past 20 years, silicon photonics has emerged as a pivotal technology in numerous integrated photonics applications, offering a mature platform for constructing large-scale integrated optoelectronic devices [1]. However, the challenge of effectively coupling light into and out of silicon photonic components persists, largely due to the significant mismatch in size between the mode distributions of optical fibers and silicon photonic waveguides [2]. Generally, two coupling methods are adopted to couple light between optical fibers and photonic waveguides: horizontal edge couplers and vertical surface couplers. Compared to other types of couplers, grating couplers that use the vertical diffraction light field of on-chip optical waveguides have the advantages of wafer scale testability, large alignment tolerance, and placement flexibility, making them a research focus in the field of photonic integration [3]. Extensive research has been dedicated to silicon-based grating couplers for efficient fiber-to-chip coupling, taking advantage of the CMOS compatibility of silicon and the high index contrast with SiO_2_ cladding for optimal optical confinement [4]. Yet, the inherent properties of silicon, such as its substantial thermo-optic coefficient, make the performance of Silicon-On-Insulator (SOI) devices highly sensitive to temperature variations [1], which poses limitations for their use in all-optical processing applications. On the contrary, Si_3_N_4_ has gained significant attention for the realization of integrated photonic devices, due to its CMOS compatibility, exceptionally low propagation losses, negligible two-photon absorptions in the C-band, reliable temperature stability, and suitability for a wider working wavelength range, including the visible spectrum [5]. Generally, Si_3_N_4_-based grating couplers achieve a larger bandwidth compared to their silicon-based counterparts [6]. However, they often suffer from a lower coupling efficiency due to the relatively low refractive index of the material, which restricts the scattering strength and directionality of the grating, making it particularly difficult to match the optical intensity profile between the mode distributions of optical fibers and photonic waveguides [7].

During the past two decades, Si_3_N_4_ has proven to be a valuable asset in heterointegration platforms, enhancing additional functionalities [8,9,10]. Therefore, the integration of high-performance Si_3_N_4_ waveguides with SOI on the same platform, fully leveraging their strengths, is becoming an important direction in photonics integration. Jason et al. designed silicon nitride-on-silicon bi-layer grating couplers using an optimization-based procedure and obtained a peak coupling efficiency of −2.2 dB with a 1-dB bandwidth of 72.9 nm [11]. Sacher et al. developed grating couplers with aligned Si_3_N_4_ and silicon grating, achieving significant bandwidths and high coupling efficiencies, that is, a reported maximum coupling efficiency of −1.3 dB and a 1-dB bandwidth of 80 nm [12]. However, the lack of a bottom reflector limits the efficiency of these couplers. Xu et al. successfully fabricated high-efficiency apodized grating couplers, achieving a maximum coupling efficiency of −2.5 dB and a 1-dB bandwidth of 65 nm [7]. Chmielak et al. proposed and demonstrated an amorphous silicon layer on top of silicon nitride to improve the directionality of the coupler, obtaining a coupling loss of 5 dB and a 3-dB bandwidth of 75 nm [13]. Furthermore, Vitali et al. numerically demonstrated the design of highly efficient dual-level silicon-Si_3_N_4_ apodized grating couplers, achieving a coupling efficiency exceeding −0.45 dB [14]. However, the fabrication of such apodized grating couplers remains complex.

The most important characteristics of an excellent grating coupler device are a high coupling efficiency and large bandwidth, and the difficulty of fabrication also needs to be considered. In this study, a uniform grating coupler with a high coupling efficiency and broad bandwidth, based on a Si_3_N_4_-on-SOI platform, is proposed and numerically demonstrated. The grating coupler consists of a bottom reflector and a coupled grating, which can improve the radiation directionality and reduce the mismatch between the mode distributions of optical fibers and photonic waveguides, respectively. Through the optimization of the grating structure, it is found that the coupling efficiency to a standard single-mode fiber reaches 81.8% (−0.87 dB) and the 1-dB bandwidth extends to 540 nm at a wavelength of 1550 nm.

## 2. Simulation Model and Numerical Method

A grating coupler consists of a periodic structure, usually realized by etching a high-refractive-index waveguide. Light deposited on the grating will diffract from the grating at an angle determined by the Bragg condition [3].

(1) $$\vec{k_{z}}=\beta +m\vec{K}$$

(2) $$\beta =k_{0}n_{eff}=\frac{2\pi }{\lambda_{0}}n_{eff}$$

where $\vec{k_{z}}$ is the z-component of the wave vector, β is the effective propagation constant of the optical mode in the grating, m is the diffraction order, grating vector $\vec{K}=\frac{2\pi }{\Lambda }$, and Λ is the period of the grating.

**Figure 1:**
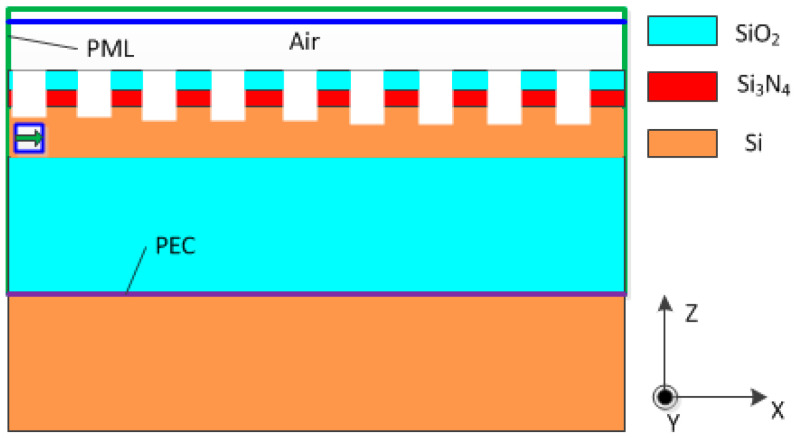
Schematic of the grating coupler based on the Si_3_N_4_-on-SOI platform.

Based on the Bragg condition, we can preliminarily calculate the period of the grating and determine the possible diffraction order and direction of the grating, but we cannot obtain the energy of each diffraction order, let alone calculate the coupling efficiency of the grating coupler. In addition, the Bragg condition does not take into account the etching depth and filling factor of the grating, which are crucial for the design of the grating couplers. Therefore, in practical design, we need to further optimize the structural design through more accurate numerical calculations and simulations, such as the finite difference time domain (FDTD). In this paper, two-dimensional (2D) FDTD numerical simulations are performed to evaluate the coupling efficiencies of the grating structures. Figure 1 presents the cross-sectional view of a grating coupler based on the Si_3_N_4_-on-SOI platform.

The grating coupler was designed using a standard SOI wafer comprising a 220 nm silicon layer (colored brown) and a buried oxide (BOX) layer (colored cyan). Subsequently, a Si_3_N_4_ layer (colored red) was deposited on the SOI wafer. An upper SiO_2_ cladding layer (colored cyan) was applied to cover the Si_3_N_4_ grating coupler. This layer was optimized not only to protect the device, but also to improve its coupling efficiency [15]. The grating was etched through both the upper SiO_2_ cladding layer and the Si_3_N_4_ layer, followed by partial etching of the silicon waveguide layer. Parameters including the grating period, fill factor, etching depth, and thicknesses of the SiO_2_ upper cladding layer, Si_3_N_4_, silicon waveguide, and SiO_2_ BOX layers were meticulously investigated to optimize the coupling efficiency.

In the simulations conducted, the input light wavelength was fixed at 1550 nm. The refractive indexes of silicon and SiO_2_ at this wavelength were set as n_Si_ = 3.48 and n_SiO2_ = 1.44, respectively, according to the data reported by Palik [16], while the refractive index for Si_3_N_4_ was selected to be n_Si3N4_ = 2.0 [14]. The background index of the air was set as 1. The design focused on transverse electric (TE) polarization and coupling to a single-mode fiber. To enhance the coupling efficiency, a back-reflector was integrated between the SiO_2_ BOX layer and the silicon substrate, improving the coupling directivity. Furthermore, a perfect electric conductor (PEC) layer [17] was used in the simulations (indicated by a purple line) to model the power reflection from the back-reflector. The TE polarized Gaussian source, depicted as a green arrow, was positioned within the silicon waveguide. It propagated through the scattering structure, causing diffraction. A frequency domain power monitor (indicated by a blue line) was strategically placed above the top surface of the grating coupler to calculate the Poynting flux of the upward electromagnetic radiation. The monitor time step was set as λ/16. Additionally, a perfectly matched layer (PML) of 500 nm was implemented along the four lateral boundaries and the top boundary of the simulated structure (indicated by a green line) [18], emulating an infinite space in a finite simulation environment. To balance precision and computational cost, a lateral calculation domain of 10 µm × 10 µm was chosen. The coupling efficiency between the fiber and waveguide was subsequently estimated as the ratio of the power in the waveguide mode exiting the grating coupler ($P_{out}$) to the total power from the light source ($P_{source}$).

(3) $$CE=P_{out}/P_{source}$$

It should be noted that, although the FDTD method is derived directly from the Maxwell equation and the process is very accurate, this method also has some shortcomings. For example, only when the mesh grid size approaches zero can the calculation results be achieved completely error-free. To solve this problem, on the one hand, we can obtain more accurate solutions by refining the mesh grid. Of course, this means that a larger computational memory and longer simulation time are required. On the other hand, quantum effects can be considered to obtain more accurate results by coupling the Maxwell equation and the Schrodinger equation [19,20]. Compared to the two methods, although the latter has a higher accuracy, considering both accuracy and convenience, we ignored quantum effects and chose to improve the calculation accuracy by refining the grid and setting the grid size to 5 nm.

## 3. Results and Discussion

This study meticulously examined the impact of various design parameters on the coupling efficiency of the grating coupler. These parameters included the thicknesses of the silicon layer, the Si_3_N_4_ layer, the SiO_2_ upper cladding layer, and the BOX layer, as well as the grating period, the filling factor, and the etching depth of the grating. In the simulations, each parameter was individually varied, while all the other parameters were kept constant, to calculate the respective coupling efficiencies. Initially, the optimization focused on the thicknesses of all the layers of the grating coupler stack [1]. Subsequently, an exhaustive investigation of the grating period, the filling factor, and the etching depth was conducted to identify the parameter settings that maximized the coupling efficiency.

### 3.1. Influences of the Si_3_N_4_ Layer Thickness on the Coupling Efficiency

**Figure 2:**
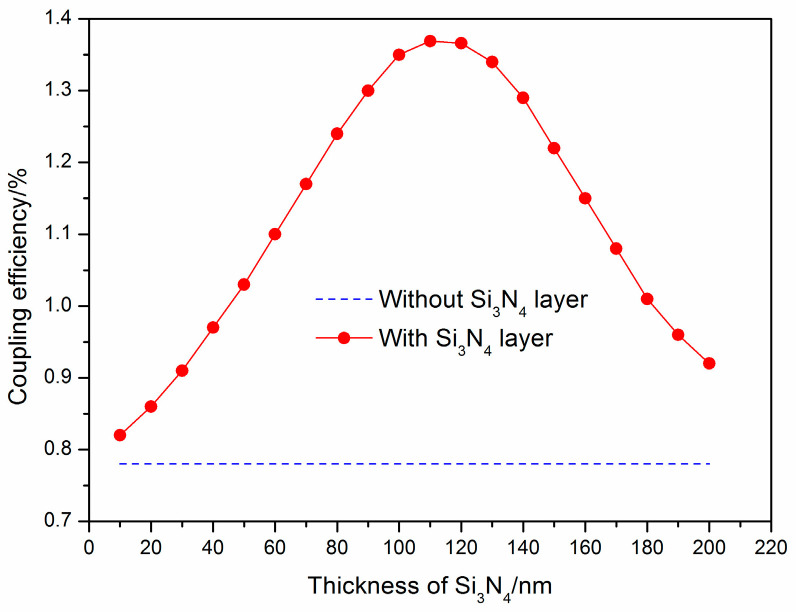
The effect of the thickness of the Si_3_N_4_ layer on the coupling efficiency.

Initially, the impact of the thickness of the Si_3_N_4_ layer on the coupling efficiency was explored. A configuration consisting of a 150 nm SiO_2_ cladding layer, a 2 µm SiO_2_ BOX layer, and a 220 nm silicon layer was selected. The thickness of the Si_3_N_4_ layer varied from 10 to 200 nm in increments of 10 nm, and the coupling efficiencies of the grating couplers were evaluated (Figure 2).

Figure 2 indicates that an increase in the thickness of the Si_3_N_4_ layer led to a corresponding increase in the coupling efficiency. However, this trend plateaued at a thickness of 110 nm, beyond which, the efficiency began to decrease. Notably, the highest coupling efficiency of 1.369% was achieved when the Si_3_N_4_ layer thickness was 110 nm.

In a grating coupler, the variation in the refractive index has a significant impact on the coupling efficiency. On the one hand, the upward diffracted light will generate Fresnel reflection on the end face of the photonic device, leading to a decrease in the coupling efficiency; on the other hand, when light is incident from the waveguide region to the grating region, there is also a problem of effective refractive index mismatch. The above two situations can be solved through the graded refractive index effect by increasing a cladding layer or changing the grating period, filling factor, and etching depth, respectively, to reduce the Fresnel reflection. Therefore, this improves the coupling efficiency. 

**Figure 3:**
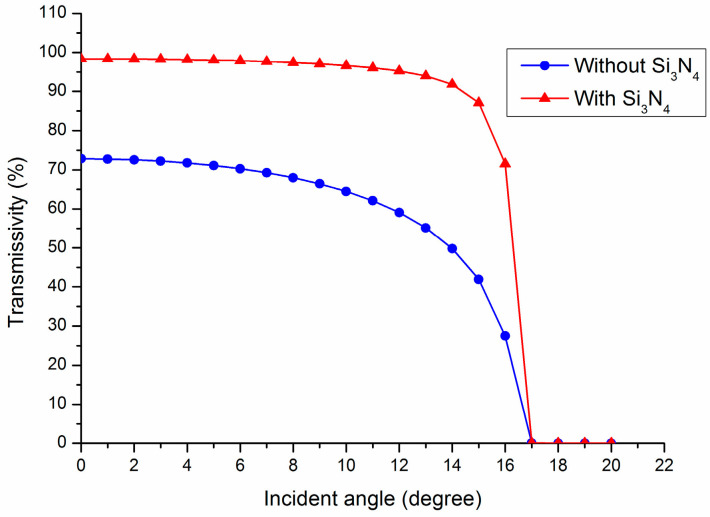
The transmissivity of couplers with and without the Si_3_N_4_ layer.

In this design, the enhancement is attributed to the graded refractive index (GRIN) effect of the layer stacks, which enables the refractive index to gradually decrease from $n_{Si}=3.48$ to $n_{Si3N4}=2$ and $n_{SiO2}=1.44$, and then to $n_{air}=1$. This can approximately satisfy the refractive index matching condition: $n_{Si3N4}^{2}\approx n_{Si}\times n_{SiO2}$ and $n_{SiO2}^{2}\approx n_{air}\times n_{Si3N4}$, which effectively suppresses the Fresnel reflection of the layer stacks [21]. In the subsequent calculations, we will further optimize the filling factor of the grating to achieve better refractive index matching, thereby reducing the Fresnel reflection and achieving a higher coupling efficiency. A 2D rigorous coupled wave analysis (RCWA) was used to calculate the total transmission efficiency of the couplers with varying Si_3_N_4_ layer thicknesses at different incident angles, as illustrated in Figure 3 [22].

Figure 3 illustrates that the transmissivity of the coupler equipped with a Si_3_N_4_ layer significantly exceeds that of the coupler without it. Specifically, the transmissivity at normal incidence for the couplers with and without a Si_3_N_4_ layer is 98.4% and 72.8%, respectively. Additionally, the average transmissivity within the extraction cone (approximately 20°) for the couplers with and without a Si_3_N_4_ layer is 76.8% and 50.8%, respectively.

Furthermore, the heterointegration of Si_3_N_4_ and SOI disrupts the vertical symmetry of the coupler. Through careful design, it is possible to achieve constructive (destructive) interference for upward (downward) radiation emanating from different interfaces.

### 3.2. Influences of SiO_2_ BOX Layer Thickness on the Coupling Efficiency

**Figure 4:**
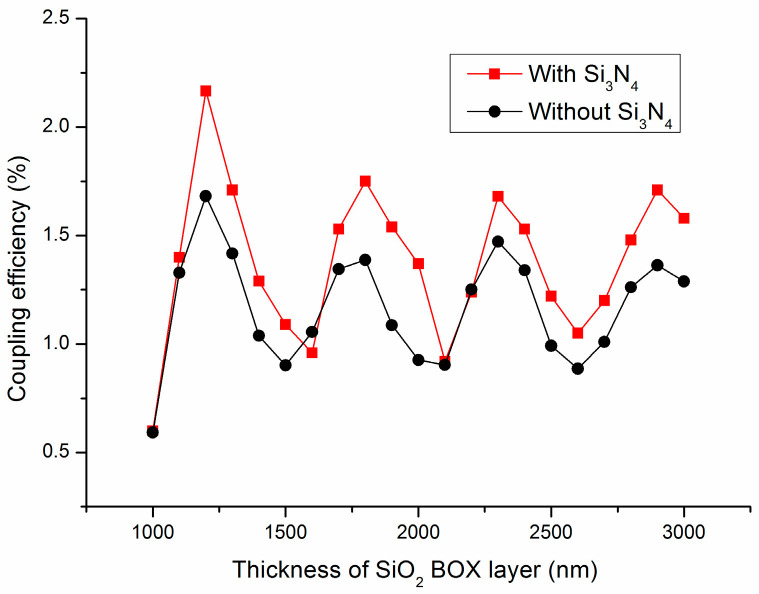
The effect of the SiO_2_ BOX layer thickness on the coupling efficiency.

Keeping the thickness of the Si_3_N_4_ layer at 110 nm, we explored the coupling efficiencies of the couplers as a function of the SiO_2_ BOX layer thickness. Beyond the intrinsic properties of the grating structure, an improvement in coupling efficiency was sought by redirecting the downward radiation power. When light was directed toward the substrate, a significant portion of the power reflected at the interface between the SiO_2_ BOX layer and the silicon substrate. This reflection could be enhanced by optimizing the thickness of the SiO_2_ BOX layer to achieve constructive interference, thereby identifying it as a crucial parameter for reflection optimization. A straightforward method to augment reflection was the insertion of a back-reflector. The coupling efficiencies of couplers with SiO_2_ BOX layer thicknesses ranging from 1000 to 3000 nm, in increments of 100 nm, were calculated, and the results are depicted in Figure 4.

Figure 4 reveals that the coupling efficiency oscillated with the increasing thickness of the SiO_2_ BOX layer [23]. Applying micro-cavity theory, the layer stack between the back-reflector and the top surface of the coupler can be conceptualized as an optical cavity, where resonances occur [24]. The optical path difference between the reflected light and upward light satisfies:

(4) $$2nd=K\frac{\lambda }{2}$$

in which n is the refractive index of the medium, λ is the wavelength of light, and K is an integer. When K is an even number, the reflected light and the directly upward emitted light undergo constructive interference and the coupling efficiency is augmented; while K is odd, the reflected light undergoes destructive interference with the direct upward outgoing light, resulting in a decrease in the coupling efficiency. As observed in Figure 4, at a SiO_2_ BOX layer thickness of 1200 nm, reflections from the substrate are in phase with the upward radiation of the coupler, resulting in a coupling efficiency of 2.167%. From Figure 4, we can also find that the thickness difference between the SiO_2_ BOX layers corresponding to the two adjacent maximum and minimum values is 700 nm, which is approximately equal to $\frac{\lambda }{2}$, and this is very in agreement with the results of the theoretical derivation. In Figure 4, we can also see that, in most cases, the coupling efficiency of the couplers with an added Si_3_N_4_ layer is higher than that without Si_3_N_4_ layers. This is precisely because the anti-Fresnel reflection effect of the Si_3_N_4_ layer analyzed above allows more optical energy to be coupled into the optical fiber.

### 3.3. Influences of SiO_2_ Cladding Layer Thickness on the Coupling Efficiency

**Figure 5:**
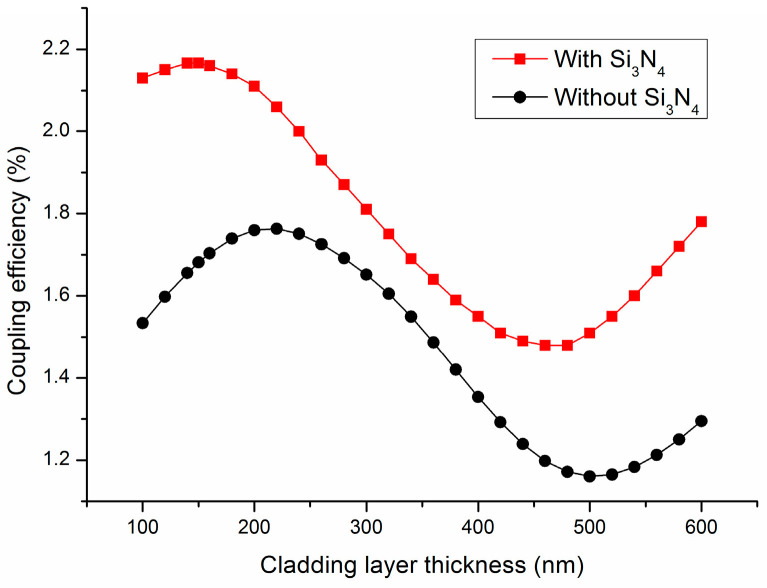
Influence of the SiO_2_ cladding layer thickness on the coupling efficiency.

As we analyzed earlier, the gradient refractive index anti-reflective layer composed of the Si_3_N_4_ layer and SiO_2_ cladding layer can effectively reduce Fresnel reflection and achieve a higher coupling efficiency. However, to achieve better anti-reflection effects, it is necessary to optimize the thickness of each layer [25]. Previously, we optimized the thickness of Si_3_N_4_, and now we will proceed with the optimization design of the SiO_2_ cladding layer thickness. With the Si_3_N_4_ layer thicknesses and the SiO_2_ BOX layer thicknesses fixed at 110 nm and 1200 nm, respectively, we investigated the coupling efficiency of the grating coupler as the SiO_2_ cladding thickness varied from 100 to 600 nm in increments of 10 nm. After a series of numerical calculations with the parameter sweep method, the results of this study are presented in Figure 5.

Figure 5 indicates that, with an increase in the SiO_2_ cladding layer thickness, the coupling efficiency increased first. However, this trend ended at a thickness of 150 nm and began to oscillate. Furthermore, when the thickness of the SiO_2_ cladding layer was 150 nm, a maximum coupling efficiency of 2.167% was achieved. This was because the 150 nm SiO_2_ cladding layer can make a more significant mode match of the waveguide facet and the single-mode fiber [26]. From Figure 5, we can also see that the double anti-reflection layer composed of Si_3_N_4_ and SiO_2_ can greatly reduce the Fresnel reflection of the coupler. For the whole structure, the SiO_2_ cladding layer mainly plays a role in matching the refractive index with the air, while the Si_3_N_4_ layer plays a significant role in the anti-reflection inside the coupler [25]. By optimizing the thickness of the Si_3_N_4_ and SiO_2_ layers, the coupling efficiency of the coupler was greatly improved. However, it is not difficult to see that, without the absence of a grating, the coupling efficiency was still very low.

### 3.4. Influences of the Grating Period and the Filling Factor on the Coupling Efficiency

**Figure 6:**
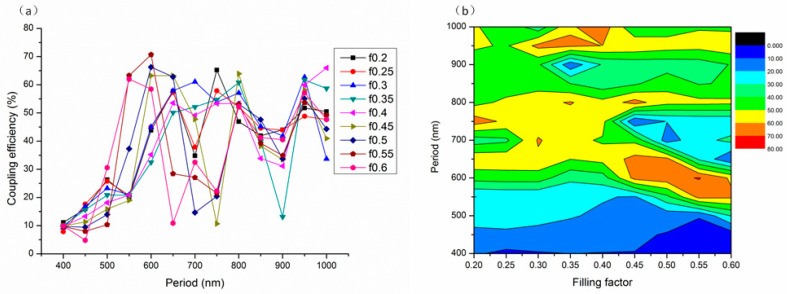
The coupling efficiency of the grating coupler with different grating periods and filling factors: (**a**) curve graph and (**b**) two-dimensional chromatogram.

The grating period and the filling factor are pivotal parameters for grating couplers. In this context, the grating period is defined as the distance between adjacent grating teeth, and the filling factor is the ratio of the top width of a grating tooth to the grating period. The study simultaneously examined the effects of the grating period and the filling factor on the coupling efficiency, given their strong interdependence. In particular, the etching depth of the silicon waveguide layer was set at 220 nm, indicating complete etching through the silicon waveguide layer, while the other parameters were maintained at their previously determined optimal values. In the simulations, the grating period varied from 400 to 1000 nm in increments of 50 nm, and the filling factor ranged from 0.2 to 0.6 in steps of 0.05. The relationship between the coupling efficiency and both the grating period and filling factor is then illustrated in Figure 6.

As shown in Figure 6a, the lines representing nine different filling factors show similar trends in coupling efficiency, with each line initially reaching a peak when the period is approximately 600 nm, followed by a zig-zag function. The coupling efficiency is significantly affected by the diffraction direction, and the diffraction angle θ is determined by:

(5) $$\Lambda n_{e}sin\theta =\Lambda n_{b}-m\lambda$$

where Λ is the grating period, $n_{e}$ is the refractive index of the environment, $n_{b}$ is the effective refractive index of the Bloch mode, m is the diffraction order, and λ is the wavelength in the vacuum. According to Equation (5), the diffraction direction is significantly determined by the grating period Λ and the effective refractive index of the Bloch mode $n_{b}$, while $n_{b}$ is determined by the etching depth and filling factor [27]. As the grating period and filling factor vary, Bloch modes diffract at different angles, resulting in oscillations in the coupling efficiency of the grating coupler, as shown in Figure 6a. However, a zig-zag function can be found as a result of only a finite number of joined points. This is very consistent with the results of the reference [28].

According to Figure 6b, the optimal period for coupling efficiency ranges between 550 nm and 650 nm, and again from 950 to 1000 nm, with the optimal filling factor lying between 0.45 and 0.6. When the grating period is set at 600 nm and the filling factor at 0.55, the phase matching condition is met, resulting in a maximum coupling efficiency of 70.69%. 

In addition, according to the effective refractive index $n_{eff}=f*n+\left(1-f\right)*n_{air}$, here, $n_{eff}$ is the effective refractive index, n is the refractive index of the dielectric materials, and $n_{air}$ is the air refractive index. We can obtain the equivalent refractive index of each layer after the grating etching: $n_{eff\_Si}=2.364$, $n_{eff\_Si3N4}=1.55$, and $n_{eff\_SiO2}=1.242$. Thus, a better refractive index matching can be obtained, further improving the coupling efficiency of the grating coupler. 

### 3.5. Effect of the Grating Depth on the Coupling Efficiency

With the grating period fixed at 600 nm and the filling factor set at 0.55, the depth of the grating varied from 280 to 480 nm in increments of 20 nm. This alteration implied that the etching depth of the silicon waveguide layer ranged from 20 to 220 nm. The corresponding coupling efficiencies of the grating coupler were then determined and analyzed.

**Figure 7:**
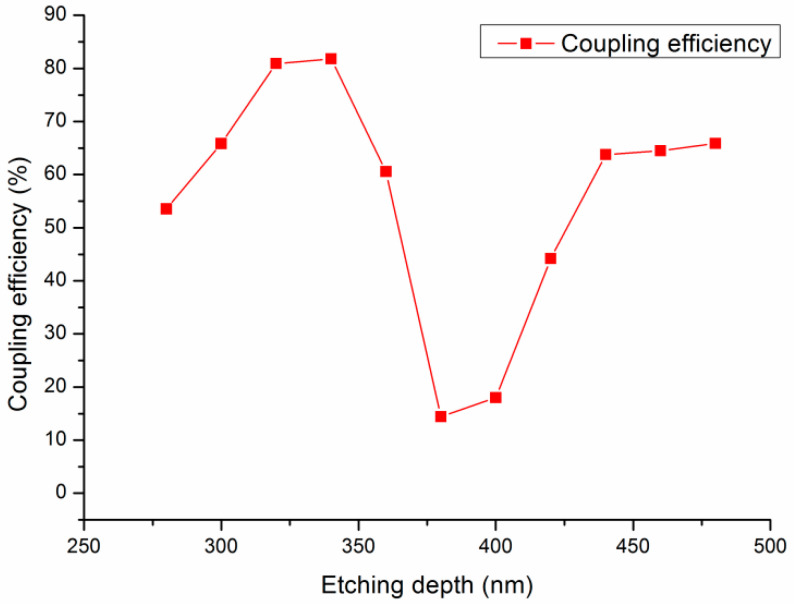
The effect of the etching depth on the coupling efficiency.

The coupling efficiencies for these varying etching depths are depicted in Figure 7. As observed in the figure, the peak coupling efficiency, reaching 81.8%, was attained when the grating depth was 340 nm, corresponding to an etching depth of 80 nm in the silicon waveguide layer.

**Figure 8:**
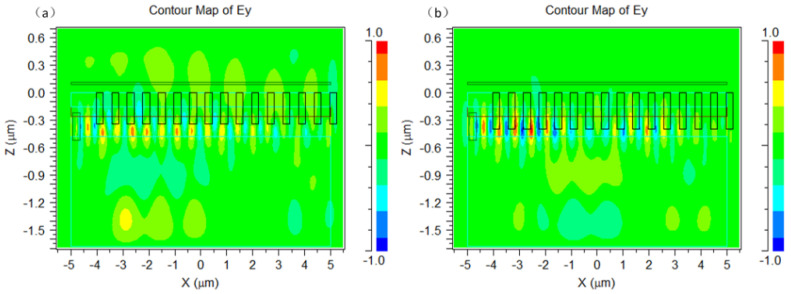
The electric field distribution of the grating coupler with etching depth equals: (**a**) 340 nm and (**b**) 400 nm.

In order to more vividly demonstrate the coupling effect of the grating coupler, we intercepted the electric field distribution of the grating coupler, which is shown in Figure 8. Figure 8 illustrates the electric field distribution in the grating coupler, with etching depths of 340 and 400 nm, respectively. It was observed that, when coupling the transverse electric fundamental mode from the left side of the silicon waveguide, the light propagated forward along the x-axis, and under the coupling effect of the grating, it diffracted upward and downward. When the etching depth was 340 nm, a significant portion of the electric field diffracted upwards, aligning well with the mode of the single-mode fiber, so as to achieve a higher diffraction efficiency. However, when the etching depth was 400 nm, most of the energy diffracted downwards from the grating, resulting in a decrease in the coupling efficiency.

### 3.6. The Wavelength Dependence of the Grating Coupler

**Figure 9:**
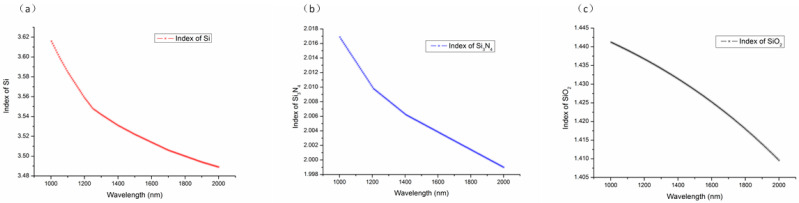
The refractive index of (**a**) silicon, (**b**) Si_3_N_4_, and (**c**) SiO_2_ with wavelengths between 1000 and 2000 nm.

In the final phase of our study, we established the grating period at 600 nm, the filling factor at 0.55, and the etching depth at 340 nm, in line with the optimal results previously obtained. We then focused on examining the variation in the coupling efficiency as the incident wavelength was altered in 10 nm increments, ranging from 1040 to 1940 nm. Given the broad spectrum of incident wavelengths, it was necessary to account for the dispersion of the materials’ refractive indices. Figure 9 presents the refractive indices of Si, Si_3_N_4_, and SiO_2_ over a wavelength range from 1000 to 2000 nm.

**Figure 10:**
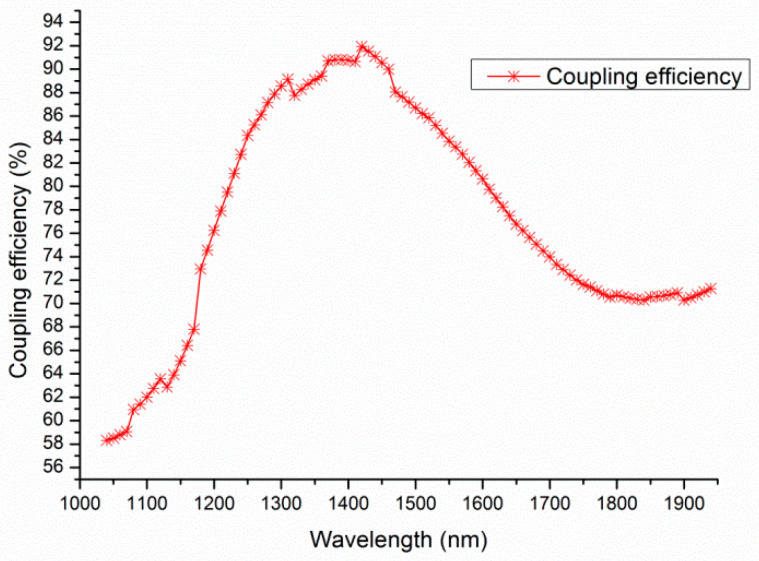
The wavelength dependency of the grating coupler.

Figure 10 shows the coupling efficiency as a function of the wavelength for the optimized Si_3_N_4_-on-SOI grating coupler developed in our study. The calculated peak coupling efficiency was 81.8% (−0.87 dB) at a wavelength of 1550 nm. Additionally, the 1-dB bandwidth of the coupler spanned 540 nm, ranging from 1180 nm to 1720 nm. This means that this grating coupler could achieve a high coupling efficiency over a wide bandwidth. The wide bandwidth was due to the relatively low refractive index of Si_3_N_4_, which allows for much fewer periods within the optical fiber [6].

For comparisons with the state of the art, Table 1 summarizes the peak coupling efficiencies and 1-dB bandwidths of different configuration grating couplers. The coupling efficiency and 1-dB bandwidth are the two most important performance indicators of grating couplers, which usually have a trade-off. Si and Si_3_N_4_, two commonly used basic materials in the fabrication of grating couplers, have complementary material properties. We can utilize the advantages of the two materials to produce grating couplers for different requirements. For the work [27], SOI-based grating couplers were fabricated to achieve a high coupling efficiency. In the work [29], Si_3_N_4_-based grating couplers were designed to obtain a larger 1-dB bandwidth; however, the coupling efficiency decreased. For the work [7,14], two materials were integrated to improve both the coupling efficiency and bandwidth. To achieve better modal matching between optical fibers and photonic devices, most grating couplers are designed as apodized gratings [7,14,27,29], and some even use double-layer structures to achieve higher mode matching and coupling efficiency [29]; however, this inevitably increases their difficulty of fabrication. The work in this article can fully utilize the material performance advantages of SOI and Si_3_N_4_, and achieved efficient wideband cost-effective uniform grating couplers without increasing the fabrication complexity. In contrast, our proposed design showed promising advantages concerning these two figure of merits.

### 3.7. The Fabrication Tolerance of the Grating Coupler

**Figure 11:**
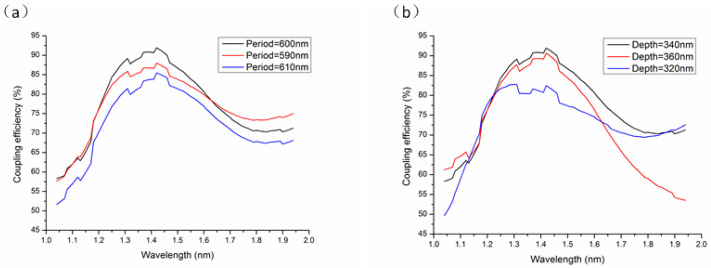
The coupling efficiency varied with the grating (**a**) period and (**b**) etching depth.

Through the above calculations, we obtained the optimal structure of the grating coupler based on the Si_3_N_4_-on-SOI platform. However, in the actual gratings’ fabrication process, due to the etching deviation, the period and depth of the gratings will inevitably bias the optimal design value to a certain extent. Thus, the effects of the fabrication tolerance on the coupling efficiency should be investigated. On that basis, we calculated the corresponding coupling efficiency of the grating coupler when there was a deviation in the grating period within the range of 10 nm and a deviation in the etching depth within the range of 20 nm, while keeping the remaining parameters unchanged. The sensitivity of the grating coupler performance on the grating period and etching depth are presented in Figure 11.

From Figure 11, it can be seen that, when the grating period and etching depth fluctuated within a relatively small range, the coupling efficiency of the grating coupler showed a similar variation trend to the optimized structure. It is indicated that an insignificant deviation in the grating configuration only exerted a weak effect on the coupling efficiency, thereby demonstrating the high fabrication tolerance of the grating coupler. These performances can be explained by the relatively low index contrast inherited by Si_3_N_4_ platforms greatly relaxing the fabrication constraints [29]. 

## 4. Conclusions

In conclusion, our research successfully proposed and demonstrated a high-efficiency, wide-bandwidth grating coupler based on Si_3_N_4_-on-SOI integrated platforms. Using Si_3_N_4_, the coupler achieved a broad bandwidth, while silicon contributed to its high efficiency. The two platforms nicely complemented each other’s shortcomings. Various configurations of the grating couplers were methodically analyzed and subsequently optimized for TE polarization. In the optimized design, we achieved a maximal coupling efficiency of 81.8% at a wavelength of 1550 nm and a 1-dB bandwidth of 540 nm. In addition, the effect of fabrication errors on the coupling performance was investigated. As revealed by the results, the proposed grating coupler had a relaxed fabrication tolerance. Future work will include a fabrication of the grating coupler according to the theoretical design and comparative tests will be conducted. This study is expected to significantly contribute to enhancing the coupling performance between silicon photonic waveguides and single-mode fibers.

## Figures and Tables

**Table 1:** Comparison of various recently reported grating couplers.

Material	Structure	Coupling Efficiency (%)	1-dB Bandwidth (nm)	Experiment/Simulation	Refs.
SOI	Apodized grating	Sim.: 83% Exp.: 81%	Exp.: 39.8 nm	Both	[27]
Si_3_N_4_	Apodized bi-layer with DBR	79.2%	117 nm	Simulation	[29]
Si–Si_3_N_4_	Dual-level Si–Si_3_N_4_ apodized grating coupler	90%	35 nm	Simulation	[14]
SiNx-on-SOI	Apodized grating coupler	56.2%	65 nm	Experiment	[7]
Si_3_N_4_-on-SOI	Uniform grating coupler with bottom reflector	81.8%	540 nm	Simulation	This work

## Data Availability

Data are contained within the article.
